# MRI Radiomic Features to Predict IDH1 Mutation Status in Gliomas: A Machine Learning Approach using Gradient Tree Boosting

**DOI:** 10.3390/ijms21218004

**Published:** 2020-10-27

**Authors:** Yu Sakai, Chen Yang, Shingo Kihira, Nadejda Tsankova, Fahad Khan, Adilia Hormigo, Albert Lai, Timothy Cloughesy, Kambiz Nael

**Affiliations:** 1 Department of Diagnostic, Molecular and Interventional Radiology, Icahn School of Medicine at Mount Sinai, New York, NY 10029, USA; yu.sakai@icahn.mssm.edu (Y.S.); chen.yang@mssm.edu (C.Y.); shingo.kihira@mountsinai.org (S.K.); 2 Department of Psychology, Cornell University, Ithaca, NY 14853, USA; 3 Department of Pathology, Molecular and Cell-Based Medicine, Icahn School of Medicine at Mount Sinai, New York, NY 10029, USA; nadejda.tsankova@mountsinai.org (N.T.); fahad.khan1@mountsinai.org (F.K.); 4 Department of Neurology, Icahn School of Medicine at Mount Sinai, New York, NY 10029, USA; adilia.hormigo@mssm.edu; 5 Department of Neurology, David Geffen School of Medicine at University of California Los Angeles, Los Angeles, CA 90095, USA; albertlai@mednet.ucla.edu (A.L.); tcloughe@ucla.edu (T.C.); 6 Department of Radiological Sciences, David Geffen School of Medicine at University of California Los Angeles, Los Angeles, CA 90095, USA

**Keywords:** glioma, radiomics, machine learning, IDH1, DWI

## Abstract

In patients with gliomas, isocitrate dehydrogenase 1 (*IDH1*) mutation status has been studied as a prognostic indicator. Recent advances in machine learning (ML) have demonstrated promise in utilizing radiomic features to study disease processes in the brain. We investigate whether ML analysis of multiparametric radiomic features from preoperative Magnetic Resonance Imaging (MRI) can predict *IDH1* mutation status in patients with glioma. This retrospective study included patients with glioma with known *IDH1* status and preoperative MRI. Radiomic features were extracted from Fluid-Attenuated Inversion Recovery (FLAIR) and Diffusion-Weighted-Imaging (DWI). The dataset was split into training, validation, and testing sets by stratified sampling. Synthetic Minority Oversampling Technique (SMOTE) was applied to the training sets. eXtreme Gradient Boosting (XGBoost) classifiers were trained, and the hyperparameters were tuned. Receiver operating characteristic curve (ROC), accuracy, and f1-scores were collected. A total of 100 patients (age: 55 ± 15, M/F 60/40); with *IDH1* mutant (*n* = 22) and *IDH1* wildtype (*n* = 78) were included. The best performance was seen with a DWI-trained XGBoost model, which achieved ROC with Area Under the Curve (AUC) of 0.97, accuracy of 0.90, and f1-score of 0.75 on the test set. The FLAIR-trained XGBoost model achieved ROC with AUC of 0.95, accuracy of 0.90, f1-score of 0.75 on the test set. A model that was trained on combined FLAIR-DWI radiomic features did not provide incremental accuracy. The results show that a XGBoost classifier using multiparametric radiomic features derived from preoperative MRI can predict *IDH1* mutation status with > 90% accuracy.

## 1. Introduction

Gliomas are primary brain tumors that account for nearly 30% of all primary brain tumors and 80% of all malignant brain tumors, and they are accountable for majority of deaths from primary brain tumors, despite advancements in treatment [1]. In patients with gliomas, those with *IDH1* mutations, specifically *IDH1 R132H*, are associated with better prognosis when compared to those with *IDH1* wildtype [2,3,4,5]. Currently, *IDH1* mutation status is identified by DNA sequencing or immunohistochemistry techniques. When considering how the World Health Organization (WHO) Classification of 2016 encourages routine testing for *IDH1* mutational status [6], noninvasive methods of glioma assessment would be highly desirable for patients.

Radiomics may have the potential of providing noninvasive assessment of *IDH1* mutational status. The study of radiomics involves the computation of an extensive number of quantitative features, referred to as “radiomic features”, which describe the imaging characteristics, such as intensity and geometry attributed to radiological images. Previous studies have utilized radiomic features to predict diagnosis, prognosis and treatment responses for patients with gliomas [7,8]. The association between radiomic features and *IDH1* genotype has also been explored [9,10].

In recent years, utilizing machine learning (ML) methods for characterizing gliomas from medical imaging have attracted attention [11]. With regards to predicting glioma characteristics from MRI radiomic features, studies have primarily explored support vector machines (SVM) and random forest (RF) classifiers [11,12]. Recently, a new open source highly scalable gradient tree boosting model named eXtreme Gradient Boosting (XGBoost) has been introduced with some promising results [13]. Whereas, RF relies on simple averaging to achieve the final ensemble, gradient boosting (GB) involves a more constructive strategy, sequentially adding models to the ensemble [14]. XGBoost is an optimized form of GB. To the best of our knowledge, no study has investigated the utility of XGBoost in identification of *IDH1* mutations in grade II, III, and IV gliomas using FLAIR and DWI radiomic features.

We hypothesized that a supervised ML approach using a XGBoost classifier would be able to predict *IDH1* mutation status purely from MRI radiomic features. Therefore, the purpose of this study was to train and optimize a XGBoost classifier with preoperative Fluid-Attenuated Inversion Recovery (FLAIR) and Diffusion-Weighted-Imaging (DWI) radiomic features and predict *IDH1* mutation status.

## 2. Results

### 2.1. Patient Characteristics

A total of 100 patients met the criteria, 60 males and 40 females. The mean ± standard deviation of age (years) was 55 ± 15. There were 19 patients with lower-grade glioma, including grade II glioma (*n* = 11) and grade III glioma (*n* = 8) and 81 patients with grade IV glioma. There were 78 *IDH1* wildtype and 22 *IDH1* mutants. Table 1 summarizes patient characteristics. Table 2 summarizes *IDH1* status and characteristics of patients within each of the training, validation, and test sets.

### 2.2. Prediction of IDH1 Mutation Status Using XGBoost Models

Our FLAR-trained XGBoost model utilized 33 final radiomic features and achieved a Receiver operating characteristic Area Under the Curve (ROC AUC) of 95%, Accuracy of 90%, Precision/Recall/f1-score of 94%/94%/94% for *IDH1* wildtype, and 75%/75%/75% for *IDH1* mutants. Of the 20 cases in the test set, it correctly classified 15 wildtype cases, incorrectly classified one wildtype as mutant, correctly classified three mutants, and incorrectly classified one mutant as wildtype.

Our DWI-trained XGBoost model utilized 71 final radiomic features and achieved ROC AUC of 97%, Accuracy of 90%, Precision/Recall/f1-score of 94%/94%/94% for *IDH1* wildtype, and 75%/75%/75% for *IDH1* mutants. Of the 20 cases in the test set, it correctly classified 15 wildtype cases, incorrectly classified one wildtype as mutant, correctly classified three mutants, and incorrectly classified 1 mutant as wildtype.

Our DWI-FLAIR trained XGBoost model utilized 88 final radiomic features and achieved ROC AUC of 91%, Accuracy of 90%, Precision/Recall/f1-score of 94%/94%/94% for *IDH1* wildtype, and 75%/75%/75% for *IDH1* mutants. Of the 20 cases in the test set, it correctly classified 15 wildtype cases, incorrectly classified one wildtype as mutant, correctly classified three mutants, and incorrectly classified one mutant as wildtype.

**Figure 1:**
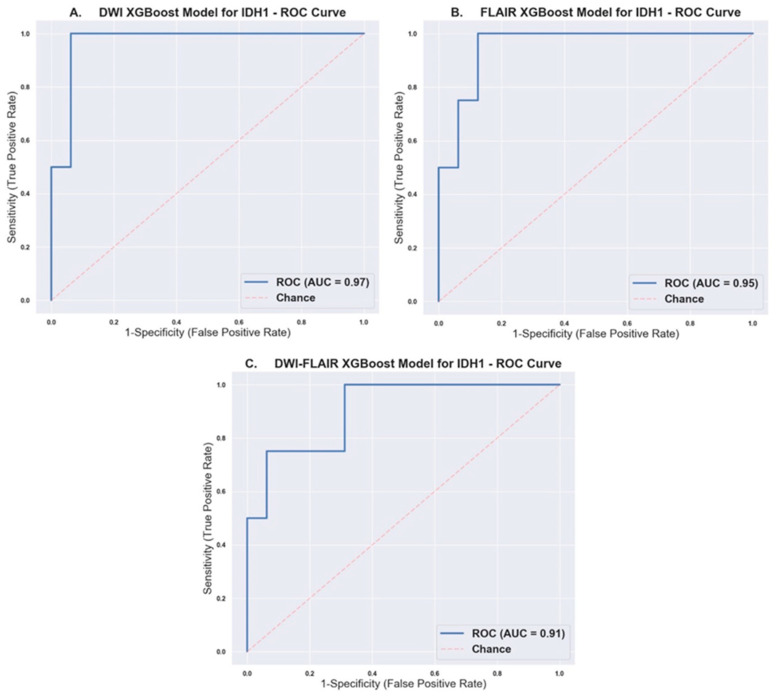
Receiver Operating Characteristic Curves with calculated AUC for (**A**) Diffusion-Weighted-Imaging (DWI), (**B**) Fluid-Attenuated Inversion Recovery (FLAIR), and (**C**) DWI-FLAIR XGBoost models.

The AUC with 95% confidence interval, Accuracy, Precision, Recall, and f1-score for each model is aggregated in Table 3. Figure 1 shows the ROC curve with the AUC score for each model.

**Figure A1:**
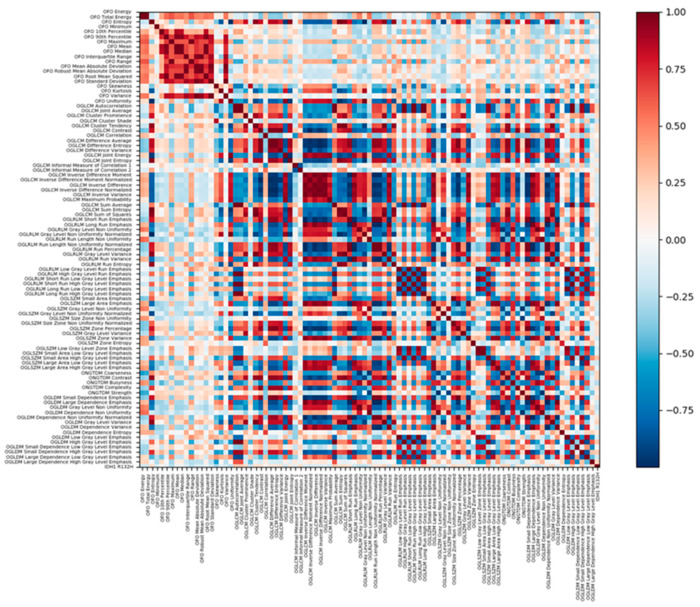
Spearman Correlation Matrix of DWI Radiomic Features.

**Figure A2:**
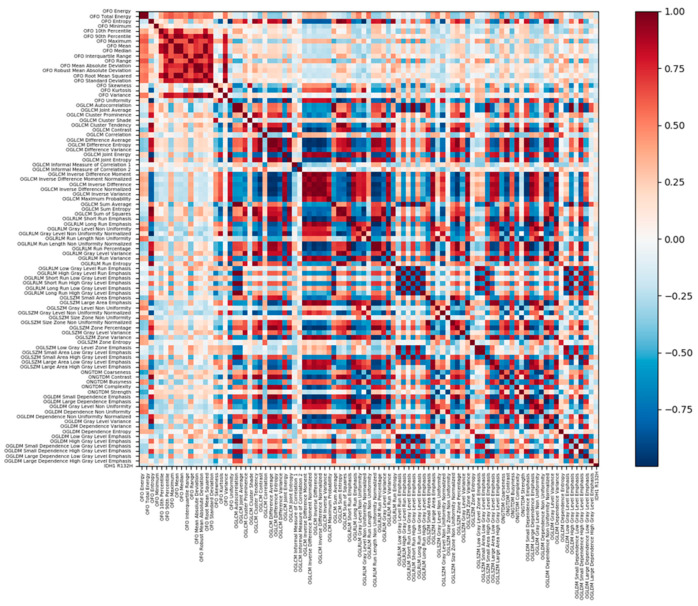
Spearman Correlation Matrix of FLAIR Radiomic Features.

The 10 most important radiomic features ordered by gain for each XGBoost model is aggregated in Table 4. Out of 184 total radiomic features, 153 were non-normally distributed (*p* < 0.05 on Shapiro–Wilks test). Total of 46 DWI and FLAIR radiomic features with significant difference between IDH1 Wildtype and Mutant are listed in Appendix A Table A1. A total of 98 DWI and FLAIR radiomic features with significant difference between glioblastomas and non-glioblastomas are listed in Appendix A Table A2. The list of final features used ranked by gain are shown in Appendix A Table A3, Table A4 and Table A5 for the DWI-FLAIR, DWI, and FLAIR XGBoost model, respectively. The list of all radiomic features by feature class are shown in Appendix A Table A6. Spearman correlation matrix of DWI and FLAIR radiomic features are shown in Appendix A Figure A1 and Figure A2.

## 3. Discussion

In patients with glioma, *IDH1* mutation has been shown to be an independent positive prognostic biomarker with improved progression-free survival and treatment outcome in comparison to the *IDH1* wildtype [15]. Although the genetic biomarkers are determined by histopathological testing, the ability to predict biomarker status noninvasively is of clinical interest, as large tissue specimens are often needed for accurate histopathological diagnosis and potential inaccuracies that are related to tumoral tissue heterogeneity. Furthermore, pre-surgical identification of these biomarkers can help in surgical planning and the determination of the extent of surgical resection.

Qualitative MRI features have been shown to correlate with *IDH1* genotypes in high grade gliomas [16,17,18]. More recently, the T2-FLAIR mismatch sign, which is defined as the presence of hyperintense signal on a T2-weighted image and a relatively hypointense signal on FLAIR (except for a hyperintense peripheral rim), has been described as a helpful imaging marker of *IDH*-mutant gliomas [19,20].

Recent improvements in ML algorithms and computational power provide an attractive venue for exploring MR radiomic features, an excellent fit for ML-approach analysis that considers the large data size and multimodal nature. Therefore, ML methods have been recently explored to predict glioma genetic biomarkers from MRI radiomic features [10,11,12,21,22,23,24,25,26,27,28,29,30,31,32,33].

Recent investigations on the use of ML and MRI-radiomics to predict *IDH1* genotype have primarily explored the SVM [21,25,30] and RF [10,22,24,27,28,29] models. Some studies only used conventional MRI sequences and achieved AUC ranging 0.84–0.96 [10,22,23,24,25,30]. Others explored the added value of advanced MRI imaging, such as MR diffusion or perfusion, with mixed results [21,26,27,28,29,31]. The highest performance of predicting *IDH1* genotype with AUC of 0.96 was observed with a RF model that was trained with conventional MRI, but this study focused only on patients with glioblastomas [10].

Expanding on above studies, we selected XGBoost as our classifier and trained models using FLAIR and DWI radiomic features, in a diverse cohort of patients, including grade II, III, and IV gliomas. Our DWI model achieved AUC of 0.97 (I: 0.898, 1.000) and 90% accuracy (Figure 1A), and our FLAIR model achieved an AUC of 0.95 (CI: 0.864, 1.000) and 90% accuracy (Figure 1B). XGBoost is a non-linear gradient boosted tree model with superior performance in comparison to conventional ML models [13]. RF and GB are both sets of decision trees. Whereas, RF builds each tree independently and combines the results at the end, GB builds each tree sequentially, and works to correct the error of the previous tree [14]. In GB, there are more hyperparameters than RF to optimize. Therefore, it may be more difficult to optimize a GB algorithm, but a better tuned GB algorithm may outperform a RF. In the training process, XGBoost calculates the importance score of each feature in each iteration, which provides a basis for establishing a new tree with gradient direction in the next iteration [13,34]. When two features are correlated, then, when deciding a split, the tree will only choose the one feature with greater importance, and this process is repeated. This automated feature selection structure is of particular use with high-dimensional data with potential multicollinearity, such as in radiomics. Another advantage of XGBoost is that it provides both L1 and L2 regularization, thus handling sparsity and reduces overfitting [13]. In this study, 92 radiomic features that were calculated by our postprocessing program were used as input to train our DWI and FLAIR classifiers, and a total of 184 radiomic features as input to train our DWI-FLAIR classifier. The DWI-FLAIR model utilized 88 out of the initial 184 features (Appendix A Table A3). The DWI model utilized 71 out of the 92 features (Appendix A Table A4). The FLAIR model utilized 33 out of the initial 92 features (Appendix A Table A5). A comparison of features that were selected by XGBoost and analysis of features with statistically significant differences between *IDH1* wildtype and mutants (Appendix A Table A1) and between glioblastomas and non-glioblastomas (Appendix A Table A2) shows the effectiveness of the automated feature selection. In review, all of the features with statistical significance between IDH1 wildtype and mutant were included in at least one of the XGBoost models, except for two features, DWI_Original Gray Level Dependence Matrix High Gray Level Emphasis and FLAIR_Original First Order Maximum. In comparison, there were seven features that had statistical significance between glioblastomas and non-glioblastomas, but they were not included in any of the XGBoost models. This is consistent with the fact that we trained the models based on *IDH1* status as opposed to glioma grade. In review of features within the top 10 that were included in our XGBoost model by automatic feature selection, but did not have statistical significant difference between *IDH1* wildtype and mutant, the mean *p*-values were 0.07 in the DWI model, 0.08 in the FLAIR model, and 0.23 in the DWI-FLAIR model. This may partially explain why we did not observe an incremental value in combining DWI and FLAIR in our XGBoost models (Figure 1C).

Our results compared favorably with the results of a recent study conducted by Shboul et al., where XGBoost models were used to predict several glioma biomarkers, including *IDH* mutation [23]. In this study, a XGBoost model was trained while using conventional MRI sequences in patients with grade II and III gliomas and reported an AUC of 0.83 in predicting *IDH* status.

In our experience, the diagnostic performance of the combined trained model using combination of DWI and FLAIR (AUC: 91%, accuracy: 90%) was comparable to the isolated model (DWI only or FLAIR only) and with no significant incremental diagnostic value (Table 3). This is likely attributed to the fact that, in the combined model, the number of features was doubled without increasing the number of observations, which can result in overfitting during the training process.

Interestingly, we observed from feature importance assessment that six out of the top 10 important radiomic features for the combined model (DWI-FLAIR) were from the DWI dataset (Table 4). DWI has shown to corelate with physiological characterization of tumors, such as cellularity and proliferation index, as a function of water diffusivity [35]. Prior histopathological studies showed that *IDH* mutations can decrease glioma proliferation through the upregulation of miR-128a [36]. It is plausible that DWI may hold invaluable information regarding the IDH status, as shown in our results. 

Our study has several limitations. Our XGBoost classifiers were trained with single-center data and, thus, the generalizability of our results may be impacted by differences in imaging acquisition protocols and the image postprocessing programs that were used for radiomic feature extraction.

The small total sample size of in combination with the skewed distribution of *IDH1* wildtype and mutant is a notable limitation. We utilized stratified sampling when splitting our dataset into train, validation, and test sets in order to account for the small proportion of *IDH1* mutants and the sampling error that could be introduced during randomization. Subsequently, as suggested by prior reports [10,32], we applied SMOTE on our train set in order to prevent biased training that favors the majority class. During our hyperparameter tuning step, the parameters were optimized for the highest ROC AUC score, as ROC curves are mathematically insensitive to class distribution unlike accuracy. Nonetheless, the effect of the skewed distribution is observed in our study (Table 2). The test set had 16 wildtype and four mutants. Our XGBoost models correctly classified 15/16 as wildtype, and correctly classified 3/4 as mutants. As the denominator is four for the mutants, one error results in a numerically steep decrease (Table 3). The skewed distribution of *IDH1* genotypes have been acknowledged in literature; studies with low grade gliomas involved predominantly *IDH1* mutants [33,37] and studies with glioblastomas involved predominantly *IDH1* wildtypes [10,28]. This is reflected in our dataset, as 17 out of 19 (89%) low grade gliomas were *IDH1* mutants and 76 out of 81 (94%) grade IV gliomas were wildtype (Table 2).

Another limitation is that, within our IDH1 wildtype population, there were two cases with minor *IDH2* (p.R172M, p.R172K) mutations. *IDH2* mutations are much less common than *IDH1* and they are mutually exclusive with *IDH1* mutations [3]. To our knowledge, there is no study that has specifically compared the radiomic features between *IDH1* and *IDH2* mutants in gliomas.

Another limitation is that tumor segmentation to generate VOI was performed by one observer and under supervision of another board-certified neuroradiologist, who made necessary adjustments before the extraction of radiomic features. Therefore, inter-observer variability assessment was not performed.

Another limitation of our ML-approach is that it does not fully explain the physiological significance of radiomic features. The prolonged survival of patients with *IDH1* mutations has previously been proposed to be associated with less aggressive biological behavior from the perspective of MRI tumor heterogeneity [16]. In this study, no additional categorical variables, such as list of comorbidities, were included or analyzed in the machine learning process. Because our models were trained purely with radiomic features, future work may focus on studying the relationship between these quantitative radiomic features and tumor heterogeneity across *IDH1* genotypes.

In conclusion, training a XGBoost classifier while using multiparametric radiomic features derived from DWI and FLAIR images discriminated *IDH1* mutation status with accuracy > 90% and AUC > 0.95, which may provide an approach for noninvasive assessment of *IDH1* status in patients with gliomas. Further studies with larger and more diverse MRI datasets are required to validate and improve upon our findings.

## 4. Materials and Methods

**Figure 2:**
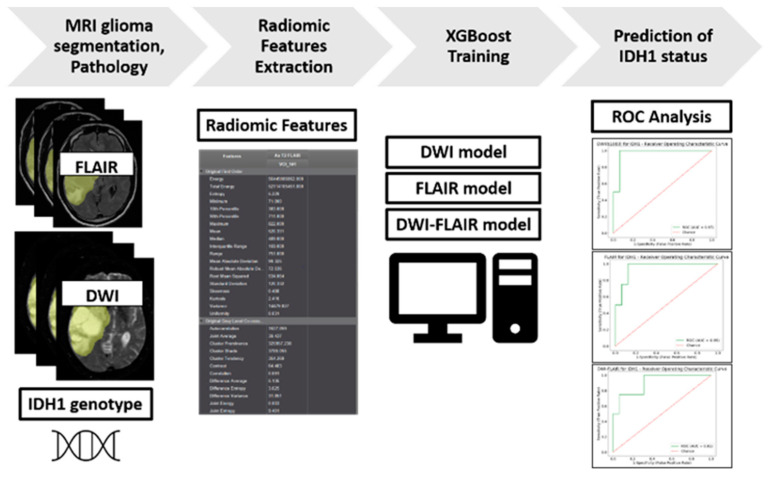
Study Design Diagram.

The overall study design is summarized as a diagram in Figure 2.

### 4.1. Patient Population

**Figure 3:**
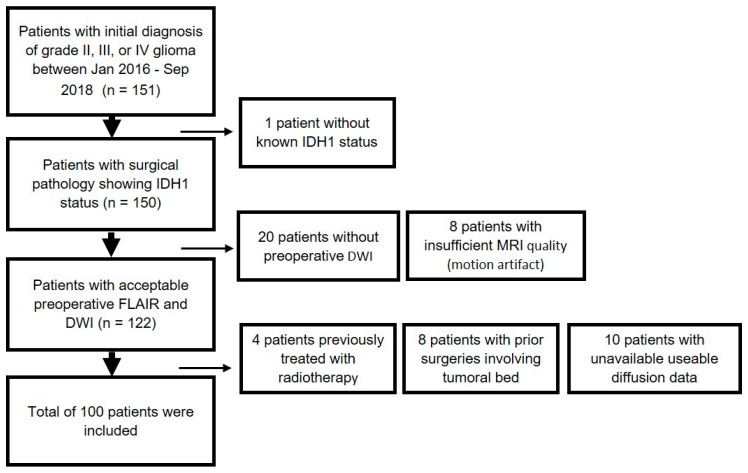
Flowchart of Patient Inclusion and Exclusion.

An institutional review board approved this retrospective study and informed consent was waived. Patients with initial diagnosis of grade II, III, or IV glioma between January 2016 to September 2018 were reviewed (*n* = 151). Patients were included if they 1) had grade II, III, or IV glioma with known IDH1 status from surgical pathology and 2) had preoperative MRI, including FLAIR, T1c+, and diffusion within 30 days of biopsy or surgical resection. One patient was removed due to lack of IDH1 status. 20 patients were excluded due to lack of preoperative DWI. The patients were excluded if they had insufficient MR image quality (motion artifact, *n* = 8). Patients were excluded if they had prior surgeries involving the tumoral bed (*n* = 8). Patients were excluded if they had prior radiotherapy treatment (*n* = 4). In addition, 10 patients were excluded due to unavailable useable diffusion data. This yielded, in total, a cohort of 100 patients (Figure 3). Assuming incidence of >15,000 new cases of malignant gliomas per year in the United States [38], 5% margin of error, 90% confidence interval, and estimated IDH mutation rate of 12% in literature [39], the recommended sample size is 114 [40]. With our sample size of 100, the margin of error is 5.33%.

### 4.2. Histopathological Data

Tissue samples were obtained from patients undergoing MRI-guided tissue biopsy or tumor resection, as part of routine clinical care and diagnostic neuropathology and molecular evaluation. Hematoxylin and eosin (H&E) sections and immunohistochemistry (IHC) slides were re-reviewed by pathologists (N.T) and (F.K). IHC was performed on 5 micron thick sections from paraffin embedded tumor sections of all the evaluated patients. Cut sections were backed in 60 °C for one hour to deparaffinize and enhance tissue adhesion; following deparaffinization, the sections were stained with pre-diluted monoclonal anti-mIDH1 antibody, purified from culture supernatant in PBS (2% BSA, 0.05% NaN3, pH 7.4, DIA-H09L; Dianova, Hamburg, Germany), which has specificity for human IDH1 R132H point mutation. The high frequency and distribution of the IDH1 R132H mutation allow the highly sensitive and specific discrimination of higher-grade gliomas by immunohistochemistry. The staining was performed using Ventana Benchmark XT stainer, (pretreated with CC1 mild, detection DAB ultraview kit; Ventana Medical Systems, Tucson, Arizona). The majority of IDH1 mutations in diffuse gliomas occur at a specific sites and they are characterized by a base exchange of guanine to adenine within codon 132, resulting in an amino acid change from arginine to histidine (R132H). Therefore, a monoclonal antibody has been developed in order to detect the consistent mutant iteration site of IDH mutant protein, allowing for its use in paraffin-embedded specimens (mIDH1R132H). The ability of the antibody to detect a small number of cells as mutant makes IHC more sensitive than sequencing for identifying R132H mutant gliomas. However, mutations in IDH2 and other mutations in IDH1 will not be detected using IHC. Next generation sequencing (NGS) was performed to confirm an IDH1 R132H negative IHC results, and/or if the patient is less than 55years old, as IDH mutations in general are extremely rare in patients over 55 years, as per College of American Pathologists (CAP) recommendations.

### 4.3. NGS Analysis

Clinical samples after the histological diagnoses of primary CNS glioma were tested for prognostic molecular biomarkers, as outlined in the NCCN guidelines, depending on the clinical and pathological context, NGS analysis was performed according to a licensed protocol in the molecular pathology lab where histological evidence of tumor cellularity of >20% was considered to be acceptable. Extracted DNA was submitted to amplicon-based library preparation and sequencing according to manufacturer’s procedures for the 50-gene Hotspot panel on a (Ion Personal Genome Machine™ (PGM™) System, Thermo Fisher Scientific Inc. Waltham, MA). In each run, a low level VAF (variant allelic frequency) control was included for both SNVs and indels. For each specimen, an average minimum coverage of 500× was considered to be acceptable to proceed. Based on the variants identified by the Torrent Variant Caller, a pipeline was applied to permit annotation, filtering of variants with a VAF of >5% (unless clinically relevant and with a VAF of >10%), and the exclusion of those in Genome Aggregation Database (gnomAD https://gnomad.broadinstitute.org/) with a population frequency >0.05%. Read depths of IDH1, and IDH2 targets were confirmed in all cases to be adequate to rule out false negatives. Molecular pathology workup of Gliomas also included NGS based testing (IHC) of Isocitrate Dehydrogenase. If, through immunohistochemistry, the IDH result was negative then, for those patients with >55 years, NGS was not performed, unless clinically indicated. For patients <55 years, NGS was performed in order to identify other IDH1 or IDH2 mutations.

### 4.4. Image Acquisition

MR imaging was obtained using seven MRI scanners (2 Skyra 3T and 2 Aera 1.5T from Siemens Healthineers, Erlangen Germany; 2 Signa 1.5T and one Discovery 3T from GE Healthcare, Waukesha, WI) within our Radiology Department. Image acquisition was performed using a standardized preoperative brain tumor MRI protocol within our radiology department, including: FLAIR (TR/TE/TI, 8000–12,000/98–130/2400–2700 ms, voxel size: 0.5 × 0.5 × 1 mm^3^), DWI (TR/TE: 4025-4600/65-82 ms, with b values of 0 and 1000 s/mm^2^, voxel size: 0.9 × 0.9 × 5.0 mm^3^), and post-contrast T1W imaging (TR/TE, 600–1800/9–19 ms, voxel size: 0.5 × 0.5 × 1 mm^3^). A total volume of 0.1 mmol/kg of gadobenate dimeglumine was intravenously injected for post-contrast T1W imaging.

### 4.5. Volume Acquisition and Texture Analysis

The tumors were manually segmented with volume-of-interest (VOI) analysis on commercially available FDA-approved software (Olea Sphere software, Olea Medical SAS, La Ciotat, France). T1c+, FLAIR and diffusion images (ADC/b1000) were coregistered on each examination using a 6-df transformation and a mutual information cost function. Subsequently, a VOI was generated while using a voxel-based signal intensity threshold method subsuming the entire region of FLAIR hyperintensity. This VOI was then overlaid onto coregistered T1c+ and diffusion datasets. The segmentation was conducted by 1 radiology resident (S.K) under supervision of an experienced board certified neuroradiologist (K.N.), who made necessary adjustments before radiomic features were extracted. One VOI was segmented from each patient.

Radiomic features were calculated from the VOIs using Olea Sphere software. A total of 92 texture features were collected: 19 first-order metrics, including the mean, standard deviation, skewness, and kurtosis, and 73 second-order metrics consisting of 23 gray level run length matrix [41], 16 gray level run length matrix [42], 15 gray level size zone matrix [43], five neighboring gray tone difference matrix [44], and 14 gray level dependence matrix [45]. Definitions and calculations of these features are explained elsewhere [46].

### 4.6. Statistical Analysis and ML

All of the statistical analysis was performed using Python. (Python 3.7; Packages: scipy v. 1.3.0; numpy 1.16.4; matplotlib 3.1.1; pandas 0.24.2; sklearn 0.21.2; imblearn 0.5.0; xgboost 0.90.)

#### 4.6.1. Radiomic Features Analysis

MRI radiomic features were tested for normality by the Shapiro–Wilks test. Wilcoxon rank sum test analysis was conducted in order to assess radiomic features with statistically significant (*p* < 0.05) difference between *IDH1* wildtype and mutants. Similarly, Wilcoxon rank sum test analysis was conducted to assess radiomic features with a statistically significant (*p* < 0.05) difference between glioblastomas and non-glioblastomas.

#### 4.6.2. ML Classifier Procedure

Input: three datasets were created as inputs for our ML methods. (1) A table consisting of FLAIR radiomic features and *IDH1* genotype for each patient. (2) A table consisting of DWI radiomic features and *IDH1* genotype for each patient. Finally, (3) a third dataset combining the DWI and FLAIR radiomic features was created. We refer to these as the (1) FLAIR dataset, (2) DWI dataset, and (3) DWI-FLAIR dataset.

Sampling: the patients were divided into train, validation, and test sets with a 60:20:20 ratio, thus resulting in 60 cases for the train set, 20 cases for the validation set, and 20 cases for the test set. Stratified random sampling was employed in this process, thus approximately maintaining the ratio of *IDH1* wildtype to mutant across the subsets to be equal to the ratio in the original dataset.

Oversampling: the Synthetic Minority Oversampling Technique (SMOTE) was applied to the train sets [47].

Training: three separate XGBoost classifiers [13] were trained: DWI, FLAIR, and DWI-FLAIR.

Hyperparameter tuning: XGBoost hyperparameters were tuned on the validation set, using exhaustive grid search (scikit-learn GridSearchCV) with five-fold cross validation. The hyperparameters were optimized for the highest receiver operating characteristic curve’s area under the curve (ROC AUC) score. The following hyperparameters and ranges for exhaustive grid search were studied: eta, 0–100 with interval of 1; max_depth, 0–100 with interval of 1; min_child_weight, 0–1 with interval of 1/500; gamma 0–1 with interval of 1/500, subsample 0–1 with interval of 1/500; colsample_by_tree, 0–1 with interval of 1/500; colsample_bylevel, 0–1 with interval of 1/500; scale_pos_weight, 0–1 with interval of 1/500; leaning_rate, 0–1 with interval of 1/500; *n*_estimators, 0–500 with interval of 1; and, reg_alpha, 0–100 with interval of 1. The details of these parameters are accessible elsewhere [48]. Three final models were collected: DWI, FLAIR, and DWI-FLAIR.

Feature importance by XGBoost: the importance of each radiomic feature was assessed and collected, ordered by the average Gain across all splits, the feature was used in. The Gain was calculated by taking each feature’s contribution for each tree in the XGBoost models and, thus, represents the relative contribution of the feature to the model.

Testing: each of the final models were tested using the respective test sets: DWI test set, FLAIR test set, and the DWI-FLAIR test set. For each model, a classification report containing the Accuracy, Precision, Recall, and f1-score was collected. For each model, the confusion matrix depicting the number of true positives, false positives, true negatives, and false negatives was collected. For each model, a ROC curve was drawn and the AUC was calculated. For each AUC, the 95% confidence interval was also calculated.

## Figures and Tables

**Table 1:** Patient Characteristics.

Patient Characteristics	*n* (%)
Total Patients	100
Female	40 (40)
Male	60 (60)
Age (in years)	
Mean	55 ± 15
Median	57
Range	28–88
Presence of enhancement on MRI	82 (82)
IDH1 status by immunohistochemistry	
Wildtype	78 (78)
Mutant	22 (22)
WHO Grade	
Grade II	11 (11)
Grade III	8 (8)
Grade IV	81 (81)

**Table 2:** IDH1 Status and Characteristics of Patients in Training/Validation/Test Sets.

Subset	IDH1 Status	Total	Male	Female	Grade II	Grade III	Grade IV
Training (*n* = 60)	Wildtype	46	25	21	1	0	45
	Mutant	14	10	4	5	5	4
Validation (*n* = 20)	Wildtype	16	10	6	0	0	16
	Mutant	4	3	1	2	1	1
Test (*n* = 20)	Wildtype	16	10	6	1	0	15
	Mutant	4	2	2	2	2	0

**Table 3:** Receiver operating characteristic Area under the Curve (ROC AUC), Accuracy, Precision, Recall, and F1-Score of Trained XGBoost Models.

Model	ROC AUC [95% CI]	Accuracy	Precision	Recall	F1-score
**DWI**	0.97 [0.898, 1.000]	0.90	Wildtype: 0.94 Mutant: 0.75	Wildtype: 0.94 Mutant: 0.75	Wildtype: 0.94 Mutant: 0.75
**FLAIR**	0.95 [0.864, 1.000]	0.90	Wildtype: 0.94 Mutant: 0.75	Wildtype: 0.94 Mutant: 0.75	Wildtype: 0.94 Mutant: 0.75
**DWI-FLAIR**	[0.741, 1.000]	0.90	Wildtype: 0.94 Mutant: 0.75	Wildtype: 0.94 Mutant: 0.75	Wildtype: 0.94 Mutant: 0.75

**Table 4:** Top 10 Most Important Radiomic Features Ranked By Gain for Each Model.

DWI-FLAIR	FLAIR	DWI
DWI_Original First Order Total Energy	Original Gray Level Run Length Matrix Short Run High Gray Level Emphasis	Original Gray Level Co-occurrence Matrix Autocorrelation
DWI_Original First Order Mean Absolute Deviation	Original First Order Mean Absolute Deviation	Original Gray Level Run Length Matrix Run Entropy
FLAIR_Original First Order 90th Percentile	Original Gray Level Co-occurrence Matrix Correlation	Original Gray Level Dependence Matrix Dependence Non Uniformity Normalized
FLAIR_Original Gray Level Dependence Matrix Small Dependence High Gray Level Emphasis	Original Gray Level Size Zone Matrix Gray Level Variance	Original Gray Level Dependence Matrix Gray Level Variance
FLAIR_Original Gray Level Run Length Matrix High Gray Level Run Emphasis	Original Gray Level Size Zone Matrix Low Gray Level Zone Emphasis	Original Gray Level Co-occurrence Matrix Maximum Probability
FLAIR_Original Gray Level Size Zone Matrix Gray Level Non Uniformity	Original Gray Level Co-occurrence Matrix Informal Measure of Correlation 2	Original Gray Level Run Length Matrix Long Run High Gray Level Emphasis
DWI_Original First Order Maximum	Original Gray Level Co-occurrence Matrix Cluster Prominence	Original Gray Level Run Length Matrix Gray Level Non Uniformity
DWI_Original Gray Level Run Length Matrix Run Entropy	Original Gray Level Dependence Matrix Small Dependence High Gray Level Emphasis	Original Gray Level Size Zone Matrix Small Area High Gray Level Emphasis
DWI_Original First Order Skewness	Original Neighboring Gray Tone Difference Matrix Coarseness	Original First Order Total Energy
DWI_Original First Order 10th Percentile	Original First Order Range	Original Gray Level Co-occurrence Matrix Informal Measure of Correlation 2

## Abbreviations

AUC	Area Under the Curve
ADC	Apparent Diffusion Coefficient
DWI	Diffusion-Weighted-Imaging
FLAIR	Fluid-Attenuated Inversion Recovery
GB	Gradient Boosting
H&E	Hematoxylin and eosin
IDH1	Isocitrate Dehydrogenase 1
IHC	Immunohistochemistry
MRI	Magnetic Resonance Imaging
NGS	Next Generation Sequencing
RF	Random Forest
ROC	Receiver Operating Characteristic curve
SMOTE	Synthetic Minority Oversampling Technique
SVM	Support Vector Machine
T1c+	Post-contrast T1
XGBoost	eXtreme Gradient Boosting

## Appendix A

**Table A1:** DWI and FLAIR radiomic features with significant difference by Wilcoxon rank sum test between IDH1 Wildtype and Mutant.

Radiomic Feature	*p*-Value	IDH1 Wildtype Mean ± SD	IDH1 Mutant Mean ± SD
FLAIR_Original Gray Level Run Length Matrix Short Run High Gray Level Emphasis	<0.001	1093.41 ± 372.49	1456.95 ± 376.78
FLAIR_Original Gray Level Size Zone Matrix Small Area High Gray Level Emphasis	<0.001	635.48 ± 187.18	824.98 ± 1 91.8
FLAIR_Original Gray Level Run Length Matrix High Gray Level Run Emphasis	<0.001	1201.22 ± 405.95	1602.17 ± 426.86
FLAIR_Original Gray Level Dependence Matrix High Gray Level Emphasis	<0.001	1209.73 ± 412.96	1613.11 ± 433.94
FLAIR_Original Gray Level Co-occurrence Matrix Cluster Shade	0.001	−167.45 ± 1997.12	−2819.29 ± 5053.17
FLAIR_Original Gray Level Co-occurrence Matrix Autocorrelation	0.001	1228.14 ± 426.63	1619.82 ± 455.56
FLAIR_Original Gray Level Co-occurrence Matrix Joint Average	0.002	33.81 ± 6.42	39.06 ± 5.76
FLAIR_Original Gray Level Co-occurrence Matrix Sum Average	0.002	67.62 ± 12.85	78.12 ± 11.52
FLAIR_Original First Order Skewness	0.003	−0.02 ± 0.57	−56 ± 0.67
DWI_Original Gray Level Size Zone Matrix Small Area High Gray Level Emphasis	0.005	546.7 ± 250.71	743.57 ± 287.76
FLAIR_Original Gray Level Run Length Matrix Long Run High Gray Level Emphasis	0.006	1997.25 ± 716.52	2621.33 ± 982.83
DWI_Original Gray Level Run Length Matrix High Gray Level Run Emphasis	0.007	831.51 ± 457.59	1205.94 ± 547.74
DWI_Original Gray Level Run Length Matrix Short Run High Gray Level Emphasis	0.007	769.52 ± 434.1	1119.01 ± 509.77
DWI_Original Gray Level Dependence Matrix High Gray Level Emphasis	0.007	825.3 ± 462.6	1201.58 ± 553.71
FLAIR_Original Gray Level Dependence Matrix Small Dependence High Gray Level Emphasis	0.007	241.62 ± 118.86	316.36 ± 117.92
DWI_Original First Order Skewness	0.008	0.46 ± 1.06	−0.32 ± 0.9
FLAIR_Original First Order Range	0.010	1214.54 ± 671.87	989.59 ± 882
DWI_Original Gray Level Run Length Matrix Long Run High Gray Level Emphasis	0.010	1211.61 ± 574.88	1710.77 ± 768.55
DWI_Original Gray Level Dependence Matrix Small Dependence High Gray Level Emphasis	0.012	213.92 ± 161.61	312.42 ± 172.48
DWI_Original Gray Level Co-occurrence Matrix Autocorrelation	0.012	821.63 ± 471.38	1184.7 ± 570.82
DWI_Original Neighboring Gray Tone Difference Matrix Busyness	0.013	1.23 ± 1.02	0.68 ± 0.53
DWI_Original First Order Maximum	0.014	1174.11 ± 596.34	835.35 ± 453.04
FLAIR_Original Neighboring Gray Tone Difference Matrix Busyness	0.014	4.04 ± 3.16	2.17 ± 1.59
DWI_Original Gray Level Co-occurrence Matrix Joint Average	0.015	26.59 ± 8.64	32.68 ± 8.43
DWI_Original Gray Level Co-occurrence Matrix Sum Average	0.015	53.18 ± 17.29	65.35 ± 16.86
DWI_Original First Order Uniformity	0.022	0.05 ± 0.02	0.04 ± 0.01
DWI_Original First Order Range	0.023	1101.2 ± 596.03	775.94 ± 454.76
DWI_Original Gray Level Dependence Matrix Gray Level Variance	0.024	62.83 ± 36.88	78.73 ± 30.73
DWI_Original Gray Level Run Length Matrix Gray Level Non Uniformity Normalized	0.024	0.05 ± 0.02	0.04 ± 0.01
DWI_Original Gray Level Run Length Matrix Gray Level Variance	0.024	64.79 ± 36.43	80.37 ± 30.18
DWI_Original Gray Level Run Length Matrix Gray Level Non Uniformity	0.027	1106.8 ± 928.02	619.32 ± 481.82
DWI_Original Gray Level Dependence Matrix Gray Level Non Uniformity	0.031	1403.34 ± 1300.29	728.69 ± 584.29
DWI_Original Neighboring Gray Tone Difference Matrix Contrast	0.032	0.1 ± 0.1	0.14 ± 0.11
DWI_Original Neighboring Gray Tone Difference Matrix Coarseness	0.034	0.00 ± 0.0015	0.0013 ± 0.0016
DWI_Original Gray Level Dependence Matrix Large Dependence High Gray Level Emphasis	0.034	15,902.19 ± 7204.39	21,277.99 ± 11,907.81
FLAIR_Original First Order Maximum	0.036	1461.66 ± 783.13	1223.18 ± 953.74
DWI_Original Gray Level Size Zone Matrix Small Area Emphasis	0.037	0.59 ± 0.05	0.62 ± 0.05
FLAIR_Original Gray Level Co-occurrence Matrix Informal Measure of Correlation 2	0.037	0.77 ± 0.09	0.82 ± 0.09
FLAIR_Original Neighboring Gray Tone Difference Matrix Strength	0.038	0.53 ± 1.08	0.51 ± 0.42
DWI_Original Gray Level Size Zone Matrix Size Zone Non Uniformity Normalized	0.039	0.33 ± 0.05	0.36 ± 0.05
FLAIR_Original Neighboring Gray Tone Difference Matrix Coarseness	0.039	0.0003 ± 0.0005	0.0003 ± 0.0002
FLAIR_Original First Order Standard Deviation	0.045	151.45 ± 77.39	132.06 ± 109.62
FLAIR_Original First Order Variance	0.045	28,850.28 ± 32,282.11	28,910.57 ± 43,752.27
FLAIR_Original First Order Total Energy	0.046	73,306,599,504.28 ± 90,074,867,196.59	78,905,231,446.5 ± 128,228,405,390.59
DWI_Original First Order Entropy	0.047	4.7 ± 0.49	4.95 ± 0.29
DWI_Original Gray Level Size Zone Matrix Gray Level Non Uniformity Normalized	0.047	0.03 ± 0.01	0.03 ± 0.005

**Table A2:** DWI and FLAIR Radiomic Features with Significant Difference by Wilcoxon Rank Sum Test between Glioblastoma and Non-glioblastoma.

Radiomic Feature	*p*-Value	Glioblastoma Mean ± SD	Non-glioblastoma Mean ± SD
FLAIR_Original Gray Level Run Length Matrix Short Run High Gray Level Emphasis	<0.001	1079.74 ± 349.33	1572.62 ± 367.53
FLAIR_Original Gray Level Size Zone Matrix Small Area High Gray Level Emphasis	<0.001	628.91 ± 172.61	882.89 ± 198.65
FLAIR_Original Gray Level Run Length Matrix High Gray Level Run Emphasis	<0.001	1187.83 ± 380.14	1722.54 ± 429.06
FLAIR_Original Gray Level Dependence Matrix High Gray Level Emphasis	<0.001	1195.65 ± 386.56	1736.83 ± 435.68
FLAIR_Original Gray Level Co-occurrence Matrix Autocorrelation	<0.001	1212.08 ± 398.97	1750.11 ± 460.47
FLAIR_Original Gray Level Dependence Matrix Small Dependence High Gray Level Emphasis	<0.001	233.31 ± 109.06	363.58 ± 120.84
FLAIR_Original Gray Level Co-occurrence Matrix Joint Average	<0.001	33.62 ± 6.08	40.72 ± 5.82
FLAIR_Original Gray Level Co-occurrence Matrix Sum Average	<0.001	67.23 ± 12.17	81.43 ± 11.65
FLAIR_Original First Order Skewness	<0.001	−0.01 ± 0.57	−0.67 ± 0.63
FLAIR_Original First Order Range	<0.001	1265.91 ± 713.65	735.05 ± 618.31
FLAIR_Original Gray Level Co-occurrence Matrix Cluster Shade	<0.001	−219.28 ± 2123.23	−3017.05 ± 5179.56
FLAIR_Original Neighboring Gray Tone Difference Matrix Busyness	<0.001	4.1 ± 3.09	1.62 ± 1.13
FLAIR_Original First Order Standard Deviation	0.001	158.12 ± 82.19	100.57 ± 84.5
FLAIR_Original First Order Variance	0.001	31,674.63 ± 34,622.28	16,879.46 ± 34,301.7
FLAIR_Original First Order Mean Absolute Deviation	0.001	126.23 ± 67.02	78.74 ± 63.44
DWI_Original Gray Level Size Zone Matrix Small Area High Gray Level Emphasis	0.001	544.01 ± 249.55	786.15 ± 274.83
FLAIR_Original First Order Interquartile Range	0.001	216.27 ± 124.42	128.84 ± 97.67
FLAIR_Original First Order Robust Mean Absolute Deviation	0.001	89.61 ± 50	54.64 ± 41.84
FLAIR_Original Gray Level Run Length Matrix Long Run Low Gray Level Emphasis	0.001	0.0031 ± 0.0049	0.0022 ± 0.0031
DWI_Original Gray Level Run Length Matrix Short Run High Gray Level Emphasis	0.001	764 ± 428.7	1197.72 ± 498.08
DWI_Original Gray Level Dependence Matrix High Gray Level Emphasis	0.001	819.66 ± 457.07	1285.02 ± 542.64
DWI_Original Neighboring Gray Tone Difference Matrix Busyness	0.001	1.24 ± 1.01	0.55 ± 0.42
DWI_Original Gray Level Run Length Matrix High Gray Level Run Emphasis	0.001	826.14 ± 452.35	1287.92 ± 536.33
FLAIR_Original Neighboring Gray Tone Difference Matrix Strength	0.001	0.5 ± 1.05	0.64 ± 0.48
DWI_Original Gray Level Dependence Matrix Small Dependence High Gray Level Emphasis	0.001	210.01 ± 158.21	344.64 ± 170.11
DWI_Original First Order Skewness	0.002	0.46 ± 1.04	−0.46 ± 0.88
DWI_Original Gray Level Run Length Matrix Long Run High Gray Level Emphasis	0.002	1206.58 ± 570.58	1811.05 ± 762.28
FLAIR_Original First Order Maximum	0.002	1510.64 ± 821.7	976.74 ± 703.15
DWI_Original Gray Level Co-occurrence Matrix Autocorrelation	0.002	814.72 ± 465.16	1271.48 ± 562.39
FLAIR_Original First Order Total Energy	0.002	78,837,906,831.01 ± 92,307,518,260.86	56,208,389,465.53 ± 124,951,899,957.93
FLAIR_Original First Order Energy	0.002	121,143,113,125.22 ± 174,604,200,352.47	93,788,946,398.58 ± 241,864,772,843.25
DWI_Original Gray Level Co-occurrence Matrix Joint Average	0.002	26.49 ± 8.51	34.04 ± 8.2
DWI_Original Gray Level Co-occurrence Matrix Sum Average	0.002	52.99 ± 17.02	68.07 ± 16.4
FLAIR_Original Neighboring Gray Tone Difference Matrix Coarseness	0.003	0.0002 ± 0.0004	0.0004 ± 0.0003
DWI_Original Gray Level Run Length Matrix Gray Level Non Uniformity	0.003	1119.88 ± 914.66	486.56 ± 346.9
DWI_Original Neighboring Gray Tone Difference Matrix Coarseness	0.003	0.001 ± 0.0015	0.0015 ± 0.0017
FLAIR_Original Gray Level Dependence Matrix Large Dependence Low Gray Level Emphasis	0.003	0.04 ± 0.04	0.02 ± 0.02
DWI_Original Gray Level Dependence Matrix Gray Level Non Uniformity	0.003	1415.7 ± 1279.42	569.48 ± 421.22
DWI_Original Neighboring Gray Tone Difference Matrix Contrast	0.004	0.1 ± 0.1	0.16 ± 0.11
FLAIR_Original Gray Level Run Length Matrix Long Run High Gray Level Emphasis	0.006	1989.38 ± 660.24	2753.4 ± 1121.6
DWI_Original Gray Level Size Zone Matrix Large Area Low Gray Level Emphasis	0.006	48.74 ± 148.12	1.35 ± 3.01
FLAIR_Original Gray Level Run Length Matrix Run Length Non Uniformity	0.006	78,567.04 ± 57,195.07	41,756.66 ± 37,247.69
FLAIR_Original Gray Level Size Zone Matrix Gray Level Non Uniformity	0.006	719.94 ± 511.92	400.45 ± 299.7
DWI_Original Gray Level Run Length Matrix Gray Level Variance	0.007	64.37 ± 36.15	84.64 ± 28.49
FLAIR_Original Gray Level Dependence Matrix Low Gray Level Emphasis	0.007	0.002 ± 0.0037	0.0016 ± 0.0026
DWI_Original Gray Level Dependence Matrix Gray Level Variance	0.007	62.45 ± 36.62	82.85 ± 29.07
FLAIR_Original Gray Level Run Length Matrix Low Gray Level Run Emphasis	0.007	0.002 ± 0.0039	0.0016 ± 0.0026
FLAIR_Original Gray Level Dependence Matrix Dependence Non Uniformity	0.007	13,941.11 ± 9449.91	7721.06 ± 5691.44
DWI_Original First Order Uniformity	0.009	0.05 ± 0.02	0.04 ± 0.01
DWI_Original Gray Level Co-occurrence Matrix Contrast	0.009	41.76 ± 29.66	56.34 ± 27.96
DWI_Original Gray Level Size Zone Matrix Small Area Emphasis	0.009	0.59 ± 0.05	0.62 ± 0.05
FLAIR_Original Gray Level Size Zone Matrix Low Gray Level Zone Emphasis	0.009	0.0025 ± 0.0059	0.0019 ± 0.0025
DWI_Original Gray Level Run Length Matrix Gray Level Non Uniformity Normalized	0.009	0.05 ± 0.02	0.04 ± 0.01
DWI_Original Gray Level Size Zone Matrix Gray Level Non Uniformity Normalized	0.010	0.03 ± 0.01	0.03 ± 0.004
DWI_Original Gray Level Size Zone Matrix Size Zone Non Uniformity Normalized	0.010	0.33 ± 0.05	0.36 ± 0.05
FLAIR_Original Gray Level Run Length Matrix Short Run Low Gray Level Emphasis	0.011	0.0019 ± 0.0038	0.0015 ± 0.0025
FLAIR_Original Gray Level Run Length Matrix Gray Level Non Uniformity	0.011	3783.52 ± 3124.39	2072.1 ± 2363.18
DWI_Original Gray Level Co-occurrence Matrix Difference Variance	0.012	20.13 ± 12.63	26.75 ± 11.87
DWI_Original Gray Level Co-occurrence Matrix Inverse Difference Moment Normalized	0.012	0.99 ± 0.01	0.99 ± 0.01
FLAIR_Original Gray Level Run Length Matrix Run Variance	0.012	0.29 ± 0.15	0.22 ± 0.14
FLAIR_Original Gray Level Size Zone Matrix Size Zone Non Uniformity	0.012	6324.41 ± 4176.75	4061.53 ± 2756.39
DWI_Original Gray Level Size Zone Matrix Gray Level Variance	0.013	91.84 ± 27.92	111.61 ± 32.95
DWI_Original Gray Level Run Length Matrix Run Length Non Uniformity	0.013	16,416.98 ± 10,512.48	10,095.05 ± 6915.17
FLAIR_Original Gray Level Dependence Matrix Gray Level Non Uniformity	0.014	4533.69 ± 3896.27	2487.02 ± 3005.24
DWI_Original Gray Level Size Zone Matrix Large Area Emphasis	0.015	5713.84 ± 17,261.49	559.63 ± 1038.73
DWI_Original Gray Level Size Zone Matrix Zone Variance	0.015	5669.15 ± 17,179.37	543.05 ± 1027.67
DWI_Original Gray Level Co-occurrence Matrix Cluster Shade	0.017	657.55 ± 2804.84	−1369.51 ± 3597.59
DWI_Original First Order Maximum	0.018	1161.89 ± 588.37	833.97 ± 489.5
DWI_Original First Order Entropy	0.019	4.7 ± 0.48	5 ± 0.27
FLAIR_Original Gray Level Run Length Matrix Long Run Emphasis	0.020	1.67 ± 0.3	1.52 ± 0.3
DWI_Original Gray Level Dependence Matrix Dependence Non Uniformity	0.022	2986.94 ± 1884.99	1937.84 ± 1175.1
DWI_Original Gray Level Dependence Matrix Small Dependence Emphasis	0.022	0.21 ± 0.09	0.26 ± 0.09
DWI_Original Gray Level Size Zone Matrix Gray Level Non Uniformity	0.024	158.2 ± 101.68	101.38 ± 57.73
DWI_Original Gray Level Co-occurrence Matrix Cluster Prominence	0.027	203,134.01 ± 165,799.16	285,344.44 ± 193,348.11
DWI_Original Gray Level Co-occurrence Matrix Maximum Probability	0.028	0.02 ± 0.02	0.01 ± 0.01
DWI_Original First Order Range	0.029	1089.95 ± 588.32	772.8 ± 487.35
DWI_Original Gray Level Co-occurrence Matrix Joint Energy	0.030	0.01 ± 0.01	0.0038 ± 0.0017
DWI_Original Gray Level Run Length Matrix Run Variance	0.030	0.28 ± 0.22	0.18 ± 0.1
DWI_Original Gray Level Co-occurrence Matrix Difference Average	0.031	4.08 ± 1.49	4.86 ± 1.48
DWI_Original Gray Level Size Zone Matrix Zone Percentage	0.031	0.24 ± 0.11	0.3 ± 0.11
FLAIR_Original Gray Level Size Zone Matrix Small Area Low Gray Level Emphasis	0.031	0.0016 ± 0.0041	0.0012 ± 0.0017
DWI_Original Gray Level Run Length Matrix Long Run Emphasis	0.032	1.68 ± 0.47	1.45 ± 0.21
FLAIR_Original Gray Level Size Zone Matrix Zone Entropy	0.032	7.64 ± 0.27	7.5 ± 0.28
DWI_Original Gray Level Co-occurrence Matrix Sum of Squares	0.033	62.05 ± 36.14	77.43 ± 30.25
FLAIR_Original First Order 90th Percentile	0.034	1067.34 ± 552.14	837.61 ± 620.08
DWI_Original Gray Level Run Length Matrix Short Run Emphasis	0.035	0.91 ± 0.04	0.93 ± 0.02
DWI_Original Gray Level Co-occurrence Matrix Inverse Difference Normalized	0.036	0.95 ± 0.02	0.94 ± 0.02
DWI_Original Gray Level Run Length Matrix Run Percentage	0.036	0.87 ± 0.05	0.9 ± 0.03
DWI_Original Gray Level Run Length Matrix Run Entropy	0.036	5.34 ± 0.31	5.51 ± 0.19
DWI_Original First Order Energy	0.038	52,082,170,32.98 ± 39,247,896,40.04	34,436,543,32.49 ± 34,586,473,38.81
DWI_Original Gray Level Dependence Matrix Large Dependence Emphasis	0.038	28.71 ± 19.21	19.22 ± 9.25
DWI_Original Gray Level Dependence Matrix Large Dependence Low Gray Level Emphasis	0.038	0.36 ± 0.98	0.07 ± 0.1
DWI_Original Gray Level Run Length Matrix Run Length Non Uniformity Normalized	0.039	0.79 ± 0.07	0.83 ± 0.05
DWI_Original Gray Level Dependence Matrix Large Dependence High Gray Level Emphasis	0.041	15,954.41 ± 7160.01	21,904.17 ± 12,525.77
DWI_Original Neighboring Gray Tone Difference Matrix Complexity	0.042	4919.68 ± 1476.01	5651.97 ± 1458.46
DWI_Original Gray Level Dependence Matrix Dependence Non Uniformity Normalized	0.043	0.14 ± 0.04	0.16 ± 0.05
DWI_Original Gray Level Co-occurrence Matrix Sum Entropy	0.046	5.55 ± 0.48	5.8 ± 0.3
DWI_Original Gray Level Co-occurrence Matrix Difference Entropy	0.049	3.36 ± 0.45	3.58 ± 0.35

**Table A3:** Final Features Used by DWI-FLAIR XGBoost Model Ranked By Gain.

Radiomic Features (*n* = 88)
DWI_Original First Order Total Energy
DWI_Original First Order Mean Absolute Deviation
FLAIR_Original First Order 90th Percentile
FLAIR_Original Gray Level Dependence Matrix Small Dependence High Gray Level Emphasis
FLAIR_Original Gray Level Run Length Matrix High Gray Level Run Emphasis
FLAIR_Original Gray Level Size Zone Matrix Gray Level Non Uniformity
DWI_Original First Order Maximum
DWI_Original Gray Level Run Length Matrix Run Entropy
DWI_Original First Order Skewness
DWI_Original First Order 10th Percentile
DWI_Original First Order 90th Percentile
FLAIR_Original First Order Total Energy
DWI_Original Gray Level Size Zone Matrix Large Area Emphasis
DWI_Original Gray Level Size Zone Matrix Zone Variance
DWI_Original Gray Level Co-occurrence Matrix Informal Measure of Correlation 2
FLAIR_Original Gray Level Co-occurrence Matrix Joint Entropy
DWI_Original Gray Level Co-occurrence Matrix Correlation
DWI_Original Gray Level Co-occurrence Matrix Contrast
FLAIR_Original Gray Level Run Length Matrix Long Run Low Gray Level Emphasis
DWI_Original Gray Level Co-occurrence Matrix Cluster Shade
FLAIR_Original Gray Level Co-occurrence Matrix Cluster Shade
DWI_Original Gray Level Co-occurrence Matrix Joint Entropy
DWI_Original Gray Level Size Zone Matrix Small Area High Gray Level Emphasis
DWI_Original Neighboring Gray Tone Difference Matrix Complexity
FLAIR_Original Gray Level Co-occurrence Matrix Joint Average
DWI_Original Gray Level Dependence Matrix Small Dependence Emphasis
FLAIR_Original Gray Level Co-occurrence Matrix Informal Measure of Correlation 1
DWI_Original Gray Level Size Zone Matrix Zone Entropy
FLAIR_Original First Order Energy
DWI_Original First Order Kurtosis
FLAIR_Original Gray Level Co-occurrence Matrix Sum Average
DWI_Original Gray Level Dependence Matrix Small Dependence High Gray Level Emphasis
FLAIR_Original First Order 10th Percentile
DWI_Original First Order Variance
FLAIR_Original Gray Level Size Zone Matrix Size Zone Non Uniformity
FLAIR_Original Gray Level Size Zone Matrix Gray Level Variance
FLAIR_Original First Order Skewness
FLAIR_Original Gray Level Dependence Matrix Dependence Non Uniformity
FLAIR_Original Gray Level Size Zone Matrix Small Area High Gray Level Emphasis
FLAIR_Original Gray Level Co-occurrence Matrix Informal Measure of Correlation 2
FLAIR_Original First Order Standard Deviation
FLAIR_Original Gray Level Size Zone Matrix Large Area Low Gray Level Emphasis
FLAIR_Original Gray Level Co-occurrence Matrix Sum of Squares
DWI_Original First Order Energy
FLAIR_Original Gray Level Co-occurrence Matrix Maximum Probability
FLAIR_Original Gray Level Co-occurrence Matrix Correlation
DWI_Original Gray Level Dependence Matrix Gray Level Non Uniformity
FLAIR_Original First Order Range
FLAIR_Original Gray Level Co-occurrence Matrix Cluster Tendency
FLAIR_Original Gray Level Dependence Matrix Large Dependence Low Gray Level Emphasis
DWI_Original First Order Entropy
DWI_Original First Order Uniformity
FLAIR_Original First Order Interquartile Range
DWI_Original Gray Level Dependence Matrix Dependence Entropy
FLAIR_Original Gray Level Dependence Matrix Large Dependence High Gray Level Emphasis
DWI_Original Gray Level Dependence Matrix Gray Level Variance
FLAIR_Original Gray Level Dependence Matrix Dependence Entropy
DWI_Original Gray Level Run Length Matrix Gray Level Variance
DWI_Original First Order Robust Mean Absolute Deviation
DWI_Original First Order Median
FLAIR_Original Gray Level Co-occurrence Matrix Difference Variance
FLAIR_Original Gray Level Run Length Matrix Gray Level Variance
FLAIR_Original First Order Minimum
FLAIR_Original Gray Level Run Length Matrix Long Run High Gray Level Emphasis
DWI_Original First Order Standard Deviation
DWI_Original Gray Level Size Zone Matrix Gray Level Non Uniformity Normalized
DWI_Original Gray Level Run Length Matrix Low Gray Level Run Emphasis
FLAIR_Original Gray Level Dependence Matrix Dependence Variance
DWI_Original Neighboring Gray Tone Difference Matrix Contrast
DWI_Original Gray Level Co-occurrence Matrix Inverse Difference
DWI_Original Gray Level Dependence Matrix Large Dependence Low Gray Level Emphasis
FLAIR_Original Gray Level Size Zone Matrix Gray Level Non Uniformity Normalized
DWI_Original Gray Level Run Length Matrix Run Variance
DWI_Original Gray Level Dependence Matrix Low Gray Level Emphasis
FLAIR_Original Gray Level Size Zone Matrix Zone Percentage
FLAIR_Original Gray Level Size Zone Matrix Small Area Low Gray Level Emphasis
DWI_Original Gray Level Co-occurrence Matrix Inverse Variance
DWI_Original First Order Minimum
DWI_Original Gray Level Co-occurrence Matrix Maximum Probability
DWI_Original Gray Level Size Zone Matrix Large Area Low Gray Level Emphasis
DWI_Original Gray Level Co-occurrence Matrix Joint Energy
FLAIR_Original Neighboring Gray Tone Difference Matrix Complexity
FLAIR_Original Gray Level Co-occurrence Matrix Contrast
FLAIR_Original Gray Level Run Length Matrix Run Entropy
FLAIR_Original Gray Level Co-occurrence Matrix Joint Energy
DWI_Original Gray Level Run Length Matrix Run Percentage
DWI_Original Gray Level Co-occurrence Matrix Joint Average
FLAIR_Original First Order Mean Absolute Deviation

**Table A4:** Final Features Used by DWI XGBoost Model Ranked By Gain.

Radiomic Features (*n* = 71)
Original Gray Level Co-occurrence Matrix Autocorrelation
Original Gray Level Run Length Matrix Run Entropy
Original Gray Level Dependence Matrix Dependence Non Uniformity Normalized
Original Gray Level Dependence Matrix Gray Level Variance
Original Gray Level Co-occurrence Matrix Maximum Probability
Original Gray Level Run Length Matrix Long Run High Gray Level Emphasis
Original Gray Level Run Length Matrix Gray Level Non Uniformity
Original Gray Level Size Zone Matrix Small Area High Gray Level Emphasis
Original First Order Total Energy
Original Gray Level Co-occurrence Matrix Informal Measure of Correlation 2
Original Gray Level Size Zone Matrix Large Area High Gray Level Emphasis
Original Gray Level Size Zone Matrix Size Zone Non Uniformity
Original First Order 10th Percentile
Original First Order Root Mean Squared
Original Gray Level Co-occurrence Matrix Cluster Shade
Original First Order Kurtosis
Original Gray Level Size Zone Matrix Low Gray Level Zone Emphasis
Original Gray Level Run Length Matrix Run Length Non Uniformity
Original Neighboring Gray Tone Difference Matrix Coarseness
Original First Order Mean Absolute Deviation
Original First Order Skewness
Original Gray Level Run Length Matrix Short Run Low Gray Level Emphasis
Original Gray Level Co-occurrence Matrix Sum Average
Original First Order Robust Mean Absolute Deviation
Original Gray Level Dependence Matrix Dependence Non Uniformity
Original First Order Range
Original Neighboring Gray Tone Difference Matrix Busyness
Original First Order Maximum
Original Gray Level Co-occurrence Matrix Sum of Squares
Original Gray Level Co-occurrence Matrix Inverse Difference Normalized
Original First Order Standard Deviation
Original Gray Level Dependence Matrix Gray Level Non Uniformity
Original Neighboring Gray Tone Difference Matrix Strength
Original Gray Level Size Zone Matrix Size Zone Non Uniformity Normalized
Original Gray Level Dependence Matrix Dependence Entropy
Original Gray Level Size Zone Matrix Zone Entropy
Original Gray Level Dependence Matrix Low Gray Level Emphasis
Original Gray Level Co-occurrence Matrix Sum Entropy
Original Gray Level Size Zone Matrix Gray Level Non Uniformity Normalized
Original Gray Level Co-occurrence Matrix Difference Entropy
Original Gray Level Co-occurrence Matrix Informal Measure of Correlation 1
Original Gray Level Run Length Matrix Long Run Emphasis
Original Gray Level Run Length Matrix Long Run Low Gray Level Emphasis
Original First Order Minimum
Original Gray Level Run Length Matrix Low Gray Level Run Emphasis
Original Gray Level Size Zone Matrix Zone Percentage
Original Gray Level Co-occurrence Matrix Cluster Prominence
Original First Order Energy
Original Neighboring Gray Tone Difference Matrix Complexity
Original Gray Level Co-occurrence Matrix Correlation
Original Gray Level Co-occurrence Matrix Joint Energy
Original Gray Level Size Zone Matrix Gray Level Non Uniformity
Original Gray Level Size Zone Matrix Zone Variance
Original Gray Level Size Zone Matrix Small Area Emphasis
Original Gray Level Run Length Matrix High Gray Level Run Emphasis
Original Gray Level Run Length Matrix Gray Level Non Uniformity Normalized
Original Gray Level Co-occurrence Matrix Difference Average
Original First Order Median
Original First Order Uniformity
Original Gray Level Co-occurrence Matrix Joint Entropy
Original Gray Level Co-occurrence Matrix Joint Average
Original First Order Mean
Original First Order Interquartile Range
Original Gray Level Size Zone Matrix Large Area Low Gray Level Emphasis
Original Gray Level Size Zone Matrix Gray Level Variance
Original Neighboring Gray Tone Difference Matrix Contrast
Original Gray Level Run Length Matrix Short Run High Gray Level Emphasis
Original Gray Level Run Length Matrix Gray Level Variance
Original Gray Level Run Length Matrix Run Percentage
Original Gray Level Dependence Matrix Large Dependence High Gray Level Emphasis
Original Gray Level Co-occurrence Matrix Contrast

**Table A5:** Final Features Used by FLAIR XGBoost Model Ranked By Gain.

Radiomic Features (*n* = 33)
Original Gray Level Run Length Matrix Short Run High Gray Level Emphasis
Original First Order Mean Absolute Deviation
Original Gray Level Co-occurrence Matrix Correlation
Original Gray Level Size Zone Matrix Gray Level Variance
Original Gray Level Size Zone Matrix Low Gray Level Zone Emphasis
Original Gray Level Co-occurrence Matrix Informal Measure of Correlation 2
Original Gray Level Co-occurrence Matrix Cluster Prominence
Original Gray Level Dependence Matrix Small Dependence High Gray Level Emphasis
Original Neighboring Gray Tone Difference Matrix Coarseness
Original First Order Range
Original Gray Level Size Zone Matrix Size Zone Non Uniformity
Original First Order Kurtosis
Original First Order Skewness
Original Gray Level Run Length Matrix Long Run High Gray Level Emphasis
Original First Order Interquartile Range
Original Gray Level Size Zone Matrix Gray Level Non Uniformity
Original Gray Level Run Length Matrix Gray Level Non Uniformity Normalized
Original Gray Level Co-occurrence Matrix Inverse Difference Moment Normalized
Original Gray Level Dependence Matrix Small Dependence Low Gray Level Emphasis
Original Gray Level Co-occurrence Matrix Cluster Shade
Original Gray Level Dependence Matrix Large Dependence Low Gray Level Emphasis
Original First Order Energy
Original Gray Level Co-occurrence Matrix Difference Entropy
Original Gray Level Run Length Matrix Run Entropy
Original Gray Level Run Length Matrix Low Gray Level Run Emphasis
Original Gray Level Size Zone Matrix Small Area High Gray Level Emphasis
Original Gray Level Run Length Matrix Run Percentage
Original Gray Level Co-occurrence Matrix Joint Average
Original Gray Level Run Length Matrix Long Run Emphasis
Original Gray Level Run Length Matrix Short Run Emphasis
Original First Order Robust Mean Absolute Deviation
Original Gray Level Run Length Matrix High Gray Level Run Emphasis
Original First Order Total Energy

**Table A6:** All Radiomic Features Listed By Feature Class.

Feature Class	Radiomic Features
**First Order Statistics**	Original First Order Energy
	Original First Order Total Energy
	Original First Order Entropy
	Original First Order Minimum
	Original First Order 10th Percentile
	Original First Order 90th Percentile
	Original First Order Maximum
	Original First Order Mean
	Original First Order Median
	Original First Order Interquartile Range
	Original First Order Range
	Original First Order Mean Absolute Deviation
	Original First Order Robust Mean Absolute Deviation
	Original First Order Root Mean Squared
	Original First Order Standard Deviation
	Original First Order Skewness
	Original First Order Kurtosis
	Original First Order Variance
	Original First Order Uniformity
**Gray Level Co-occurrence Matrix**	Original Gray Level Co-occurrence Matrix Autocorrelation
	Original Gray Level Co-occurrence Matrix Joint Average
	Original Gray Level Co-occurrence Matrix Cluster Prominence
	Original Gray Level Co-occurrence Matrix Cluster Shade
	Original Gray Level Co-occurrence Matrix Cluster Tendency
	Original Gray Level Co-occurrence Matrix Contrast
	Original Gray Level Co-occurrence Matrix Correlation
	Original Gray Level Co-occurrence Matrix Difference Average
	Original Gray Level Co-occurrence Matrix Difference Entropy
	Original Gray Level Co-occurrence Matrix Difference Variance
	Original Gray Level Co-occurrence Matrix Joint Energy
	Original Gray Level Co-occurrence Matrix Joint Entropy
	Original Gray Level Co-occurrence Matrix Informal Measure of Correlation 1
	Original Gray Level Co-occurrence Matrix Informal Measure of Correlation 2
	Original Gray Level Co-occurrence Matrix Inverse Difference Moment
	Original Gray Level Co-occurrence Matrix Inverse Difference Moment Normalized
	Original Gray Level Co-occurrence Matrix Inverse Difference
	Original Gray Level Co-occurrence Matrix Inverse Difference Normalized
	Original Gray Level Co-occurrence Matrix Inverse Variance
	Original Gray Level Co-occurrence Matrix Maximum Probability
	Original Gray Level Co-occurrence Matrix Sum Average
	Original Gray Level Co-occurrence Matrix Sum Entropy
	Original Gray Level Co-occurrence Matrix Sum of Squares
**Gray Level Run Length Matrix**	Original Gray Level Run Length Matrix Short Run Emphasis
	Original Gray Level Run Length Matrix Long Run Emphasis
	Original Gray Level Run Length Matrix Gray Level Non Uniformity
	Original Gray Level Run Length Matrix Gray Level Non Uniformity Normalized
	Original Gray Level Run Length Matrix Run Length Non Uniformity
	Original Gray Level Run Length Matrix Run Length Non Uniformity Normalized
	Original Gray Level Run Length Matrix Run Percentage
	Original Gray Level Run Length Matrix Gray Level Variance
	Original Gray Level Run Length Matrix Run Variance
	Original Gray Level Run Length Matrix Run Entropy
	Original Gray Level Run Length Matrix Low Gray Level Run Emphasis
	Original Gray Level Run Length Matrix High Gray Level Run Emphasis
	Original Gray Level Run Length Matrix Short Run Low Gray Level Emphasis
	Original Gray Level Run Length Matrix Short Run High Gray Level Emphasis
	Original Gray Level Run Length Matrix Long Run Low Gray Level Emphasis
	Original Gray Level Run Length Matrix Long Run High Gray Level Emphasis
**Gray Level Size Zone Matrix**	Original Gray Level Size Zone Matrix Small Area Emphasis
	Original Gray Level Size Zone Matrix Large Area Emphasis
	Original Gray Level Size Zone Matrix Gray Level Non Uniformity
	Original Gray Level Size Zone Matrix Gray Level Non Uniformity Normalized
	Original Gray Level Size Zone Matrix Size Zone Non Uniformity
	Original Gray Level Size Zone Matrix Size Zone Non Uniformity Normalized
	Original Gray Level Size Zone Matrix Zone Percentage
	Original Gray Level Size Zone Matrix Gray Level Variance
	Original Gray Level Size Zone Matrix Zone Variance
	Original Gray Level Size Zone Matrix Zone Entropy
	Original Gray Level Size Zone Matrix Low Gray Level Zone Emphasis
	Original Gray Level Size Zone Matrix Small Area Low Gray Level Emphasis
	Original Gray Level Size Zone Matrix Small Area High Gray Level Emphasis
	Original Gray Level Size Zone Matrix Large Area Low Gray Level Emphasis
	Original Gray Level Size Zone Matrix Large Area High Gray Level Emphasis
**Neighboring Gray Tone Difference Matrix**	Original Neighboring Gray Tone Difference Matrix Coarseness
	Original Neighboring Gray Tone Difference Matrix Contrast
	Original Neighboring Gray Tone Difference Matrix Busyness
	Original Neighboring Gray Tone Difference Matrix Complexity
	Original Neighboring Gray Tone Difference Matrix Strength
**Gray Level Dependence Matrix**	Original Gray Level Dependence Matrix Small Dependence Emphasis
	Original Gray Level Dependence Matrix Large Dependence Emphasis
	Original Gray Level Dependence Matrix Gray Level Non Uniformity
	Original Gray Level Dependence Matrix Dependence Non Uniformity
	Original Gray Level Dependence Matrix Dependence Non Uniformity Normalized
	Original Gray Level Dependence Matrix Gray Level Variance
	Original Gray Level Dependence Matrix Dependence Variance
	Original Gray Level Dependence Matrix Dependence Entropy
	Original Gray Level Dependence Matrix Low Gray Level Emphasis
	Original Gray Level Dependence Matrix High Gray Level Emphasis
	Original Gray Level Dependence Matrix Small Dependence Low Gray Level Emphasis
	Original Gray Level Dependence Matrix Small Dependence High Gray Level Emphasis
	Original Gray Level Dependence Matrix Large Dependence Low Gray Level Emphasis
	Original Gray Level Dependence Matrix Large Dependence High Gray Level Emphasis
